# Mineralocorticoid Receptor Antagonist Treatment for Steroid-Induced Central Serous Chorioretinopathy Patients with Continuous Systemic Steroid Treatment

**DOI:** 10.1155/2018/4258763

**Published:** 2018-07-22

**Authors:** Jin Young Kim, Ju Byung Chae, Jisoo Kim, Dong Yoon Kim

**Affiliations:** ^1^Department of Ophthalmology, Jeju National University Hospital, Jeju National University School of Medicine, Jeju, Republic of Korea; ^2^Department of Ophthalmology, Chungbuk National University Hospital, College of Medicine, Chungbuk National University, Cheongju, Republic of Korea

## Abstract

**Purpose:**

To investigate the effectiveness of mineralocorticoid receptor (MR) antagonist in patients with steroid-induced central serous chorioretinopathy (CSC).

**Methods:**

A retrospective review was conducted of steroid-induced CSC patients who were treated with the MR antagonist spironolactone 50 mg once per day for at least 1 month. The primary outcome measure was complete resolution rate of subretinal fluid (SRF) after spironolactone treatment. Secondary outcomes included central subfield thickness (CST), subfoveal choroidal thickness (SFCT), and best-corrected visual acuity (BCVA) changes after spironolactone treatment.

**Results:**

Seventeen eyes from 15 patients were included in this study. Conditions warranting chronic systemic steroid use were myasthenia gravis (6/15, 40%), glomerulonephritis (5/15, 33.3%), and organ transplantation (4/15, 26.7%). Mean symptom duration of CSC was 4.00 ± 3.04 months. After spironolactone treatment, 14 eyes (82.4%) showed complete resolution of SRF (*P* < 0.001) without discontinuation of systemic steroid. CST and BCVA were significantly improved after spironolactone treatment. SFCT was significantly decreased after spironolactone treatment. No patients experienced electrolyte imbalance after spironolactone treatment.

**Conclusion:**

MR antagonist treatment may be a therapeutic option for steroid-induced CSC patients. This treatment modality may be especially beneficial for steroid-induced CSC patients who cannot discontinue steroid medication due to systemic conditions.

## 1. Introduction

Central serous chorioretinopathy (CSC) is characterized by neurosensory retinal detachment; it is a self-limiting disease that usually has a good visual prognosis. Male sex, type A personality, abnormal coagulation and platelet aggregation, and infection with *Helicobacter pylori* are considered relevant risk factors for CSC [1–7].

Endogenous or exogenous steroid therapy is a significant risk factor for CSC [8]. Elevated serum glucocorticoid hormone levels are caused by systemic or endogenous steroids that act by binding to the glucocorticoid receptor (GR) and to the mineralocorticoid receptor (MR). MR activation by steroids leads to the expression of proteins regulating ion and water transport, resulting in the reabsorption of sodium [9, 10]. MRs are also present in retinal pigment epithelium (RPE) cells and in endothelial cells of the retinal and choroidal vessels [11]. Activation of MRs in the choroidal vessel by steroids causes choroid vessel dilation and leakage, resulting in choroidal hyperpermeability, which is known to be a major pathogenic factor in CSC [11]. Experimental studies have shown that intravitreal injection of glucocorticoid in rat eyes induces choroidal enlargement. Increased choroidal hyperpermeability due to MR activation by steroids can be blocked by MR antagonists. Therefore, MR antagonists are attracting interest as therapeutic agents for CSC; several experimental studies on the effects of MR antagonists have recently been published and have demonstrated favorable therapeutic effects of MR antagonists in CSC [12–18].

We speculate that the therapeutic effect of MR antagonists on CSC may be exaggerated in the case of steroid-induced CSC. However, there have been no studies on the therapeutic effects of MR antagonists on steroid-induced CSC. Therefore, we investigated the effectiveness of MR antagonist therapy in patients with steroid-induced CSC.

## 2. Methods

The medical records of steroid-induced CSC patients who were diagnosed and treated at Chungbuk National University Hospital, Cheongju, Korea, from March 2015 through December 2017 were reviewed. This study was approved by the Institutional Review Board of Chungbuk National University Hospital and followed the tenets of the Declaration of Helsinki. The inclusion criteria were as follows: (1) definite presence of subretinal fluid (SRF) in the fovea on spectral domain optical coherence tomography (SD-OCT) at baseline; (2) patients taking systemic steroids for more than 1 month; (3) evidence of diffuse and/or focal leakage on fluorescein angiography (FA); (4) CSC treatment by MR antagonist; and (5) no prior treatment, such as intravitreal bevacizumab injection (IVB) or half-fluence photodynamic therapy (H-PDT) for CSC. Patients with choroidal neovascularization or other macular disease, other ophthalmologic diseases that can affect vision, and active intraocular inflammation or infection were excluded.

### 2.1. Ophthalmic Examination

A complete ophthalmologic examination was performed at baseline. Best-corrected visual acuity (BCVA) was measured with a Snellen chart and converted to a logMAR scale. Medical histories, including symptom duration, concomitant medication, and other medical illness, were also collected from the medical records. Every patient underwent fundus photography, FA, and SD-OCT. A Heidelberg Retina Angiograph 2 (Heidelberg Engineering, Heidelberg, Germany) was used to obtain FA and indocyanine green angiographs (ICGA). ICGAs were used to assess choroidal hyperpermeability and to rule out choroidal neovascularization or polypoidal choroidal vasculopathy. A Spectralis ophthalmic imaging platform (Heidelberg Engineering, Heidelberg, Germany) was used to acquire SD-OCT images, and a custom 20° × 20° volume acquisition protocol was employed to obtain a set of high-speed scans from each eye. The integrated follow-up mode of the device was used to acquire scans of the same retinal areas at each visit. Central subfield thickness (CST) was automatically calculated as the average retinal thickness within a central circle having a 500 *µ*m radius. We also manually measured the subfoveal choroid thickness (SFCT) by using the enhanced depth image (EDI) OCT image [19].

During each visit, ophthalmic examinations were performed, including the BCVA measurement, applanation tonometry, slit-lamp examination, dilated fundus examination, fundus photography, and SD-OCT.

### 2.2. Mineralocorticoid Antagonist Treatment

Steroid-induced CSC patients were treated with systemic spironolactone 50 mg once per day until SRF resolved. Considering the possible side effects that may occur after the use of the drug, if the SRF did not decrease despite use of spironolactone for 1 month, the patient was regarded as a nonresponder, and drug use was discontinued. Spironolactone is a potassium-preserving diuretic and may cause medical side effects such as electrolyte abnormality [20]. We performed serum electrolyte examination before treatment and every month during treatment. If an imbalance occurred in serum electrolyte levels, spironolactone treatment was discontinued.

### 2.3. Statistical Analysis

The SPSS version 21.0 statistical software package (SPSS, Inc., Chicago, IL, USA) was used for statistical analysis. The paired *t*-test was used to compare BCVA, retinal thickness, and SFCT changes after spironolactone treatment. The McNemar test was used to compare eyes with SRF before and after spironolactone treatment. In all analyses, a value of *P* < 0.05 was considered statistically significant.

## 3. Results

### 3.1. Clinical Characteristics

Seventeen steroid-induced CSC eyes from 15 patients were included in this study. The participants comprised 10 males and 5 females with a mean age of 49.24 ± 7.70 years. Symptom duration prior to treatment and mean follow-up duration were 4.00 ± 3.04 months and 17.09 ± 6.40 months, respectively. Conditions warranting chronic systemic steroid use were myasthenia gravis (6/15, 40%), glomerulonephritis (5/15, 33.3%), and organ transplantation (4/15, 26.7%). The mean daily dose of systemic prednisolone and mean steroid use duration were 15.59 ± 5.58 mg and 8.94 ± 9.03 months, respectively (Table 1). The spironolactone dose was 50 mg per day and the mean duration of spironolactone treatment was 2.59 ± 1.19 months.

### 3.2. Optical Coherence Tomographic Findings Change after Spironolactone Treatment

**Figure 1:**
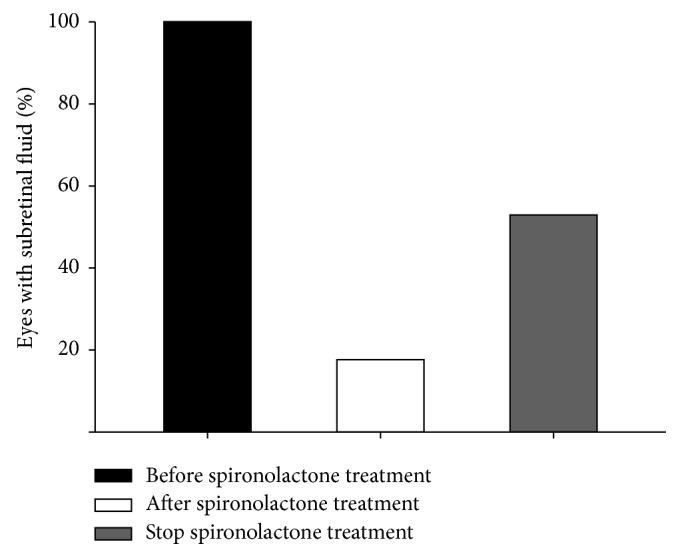
Eyes with subretinal fluid (SRF) before and after spironolactone treatment. After spironolactone treatment, 14 eyes (82.4%) showed complete resolution of the SRF (*P* < 0.001). However, among 14 eyes with complete resolution of SRF, SRF reappeared after discontinuation of spironolactone treatment in 9 eyes (64.3%, *P* < 0.001).

Figure 1 shows the percentage of eyes with SRF before and after spironolactone treatment. After spironolactone treatment, 14 eyes (82.4%) showed complete resolution of the SRF (*P* < 0.001). However, after discontinuation of spironolactone treatment, SRF reappeared in nine eyes (64.3%, *P* < 0.001). Mean time interval between discontinuation of spironolactone and recurrence was 1.55 ± 0.72 months. And, among eyes with recurrence, 6 eyes were retreated with spironolactone. After retreatment of spironolactone, SRF of 5 eyes (83.3%) was completely resolved.

**Figure 2:**
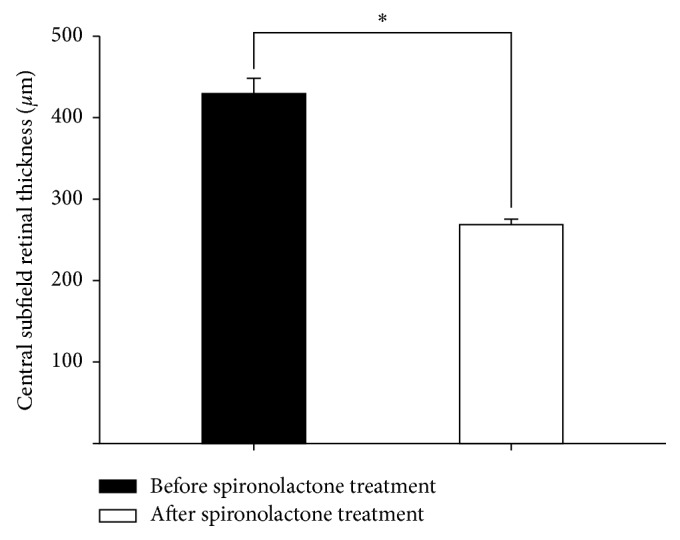
Central subfield thickness (CST) changes after spironolactone treatment. The mean CST was significantly decreased after spironolactone treatment (428.94 ± 77.70 *μ*m versus 268.47 ± 28.53 *μ*m, ^*∗*^*P* < 0.001).

**Figure 3:**
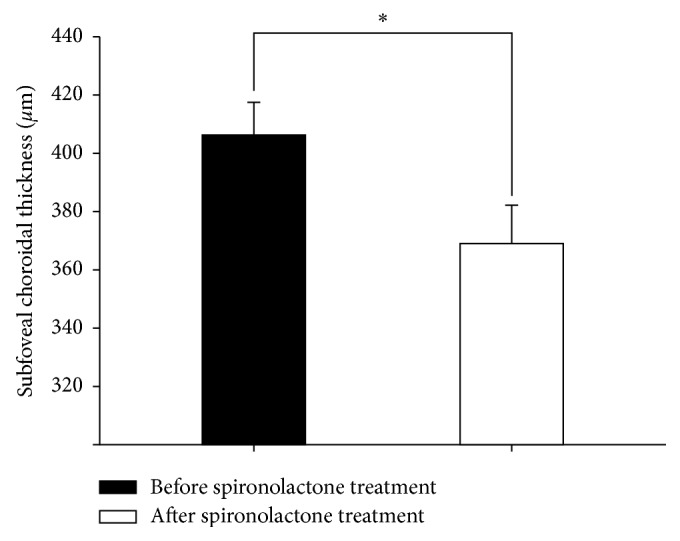
Subfoveal choroidal thickness (SFCT) changes after spironolactone treatment. The mean SFCT was significantly decreased after spironolactone treatment (406.06 ± 47.08 *μ*m versus 368.94 ± 54.29 *μ*m, ^*∗*^*P* < 0.005).

The mean CST decreased significantly after spironolactone treatment (428.94 ± 77.70 *μ*m versus 268.47 ± 28.53 *μ*m, *P* < 0.001) (Figure 2). The mean SFCT also decreased significantly after spironolactone treatment (406.06 ± 47.08 *μ*m versus 368.94 ± 54.29 *μ*m, *P*=0.034) (Figure 3).

Among 17 eyes, three eyes still showed subretinal fluid at the fovea. However, after spironolactone treatment, CST (410.00 ± 46.89 *μ*m versus 312.00 ± 14.42 *μ*m, *P*=0.035) of these 3 eyes was decreased, while the BCVA was not improved significantly (0.27 ± 0.15 versus 0.21 ± 0.20; *P*=0.188). Considering CST decrease, these three eyes could be regarded as spironolactone responder.

### 3.3. Changes in BCVA after Spironolactone Treatment

**Figure 4:**
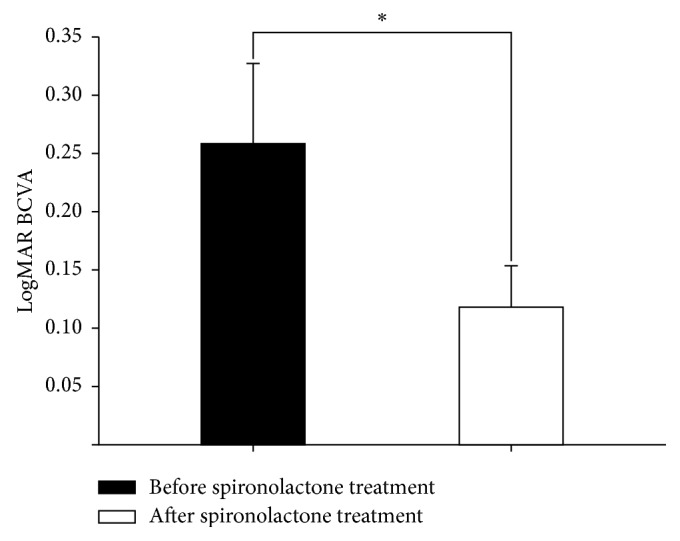
Best-corrected visual acuity (BCVA) changes after spironolactone treatment. LogMAR BCVA was significantly improved after spironolactone treatment (before, 0.280 ± 0.068; after, 0.146 ± 0.035, ^*∗*^*P*=0.021).

Figure 4 shows the BCVA changes after resolution of CSC. LogMAR BCVA significantly improved after spironolactone treatment (before, 0.280 ± 0.068; after, 0.146 ± 0.035; *P*=0.021).

**Figure 5:**
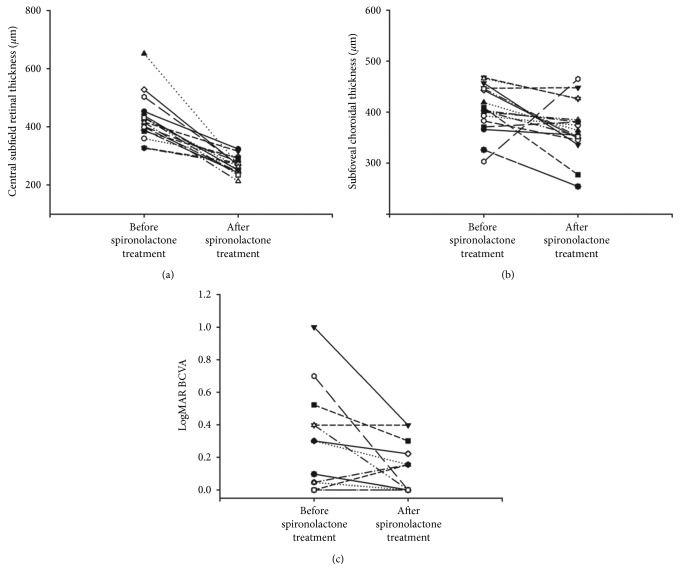
(a) Central subfield thickness (CST), (b) subfoveal choroidal thickness (SFCT), and (c) best-corrected visual acuity (BCVA) changes in each spironolactone-treated eye.

**Figure 6:**
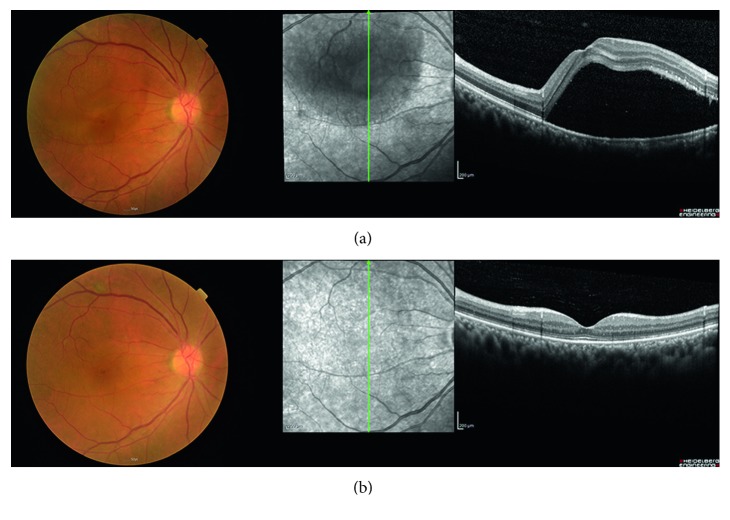
Representative case of steroid-induced CSC patient.

Figure 5 shows CST, SFCT, and BCVA change in each spironolactone-treated eye. And Figure 6 shows a representative case of steroid-induced CSC. The patient, a 59-year-old man, underwent kidney transplantation. Three months after transplantation, he was referred to the ophthalmologic clinic for visual disturbance of his right eye. Visual acuity of his right eye was 0.4, and SD-OCT examination revealed a bullous SRF (Figure 6(a)). At that time, steroid 20 mg was used to prevent graft rejection. We diagnosed steroid-induced CSC and started spironolactone treatment. Two months after spironolactone treatment, the SRF was completely resolved in the patient's right eye, and the visual acuity of his right eye improved from 0.4 to 1.0 without discontinuation of systemic steroid use (Figure 6(b)). No spironolactone treatment-related electrolyte imbalance was observed.

### 3.4. Serum Level Changes of Potassium and Creatinine after Spironolactone Treatment

Table 2 presents serum level changes in potassium and creatinine after spironolactone treatment. There were no significant changes in serum potassium or creatinine levels after spironolactone treatment. Moreover, there were no cases in which drug therapy had to be discontinued because of an electrolyte imbalance or spironolactone-related side effect.

## 4. Discussion

The primary finding of this study is that CST and BCVA significantly improved after spironolactone treatment in patients with steroid-induced CSC. In most patients, SRF was completely resolved after spironolactone treatment. However, resolved SRF recurred after discontinuation of spironolactone treatment. No patients experienced electrolyte imbalances after spironolactone treatment.

The most conventional therapeutic approach for steroid-induced CSC patients is discontinuation of regional or systemic steroids. After discontinuation of steroid use, most cases of CSC resolve without additional treatment. However, in certain patients who have received organ transplants or others requiring chronic immunosuppression, continued use of corticosteroids may be necessary, despite the presence of CSC. In these patients, recurrent or chronic CSC due to systemic steroid use can be difficult to manage and may possibly result in irreversible vision loss [8]. Argon laser photocoagulation, transpupillary thermotherapy, intravitreal injection of antivascular endothelial growth factors (VEGF), micropulse diode laser photocoagulation, and photodynamic therapy have all been used to manage steroid-induced CSC patients who cannot discontinue steroid therapy [8, 21–26]. However, to date, there has been no established treatment for steroid-induced CSC patients who cannot discontinue systemic steroid use.

Endogenous or exogenous steroid therapy is a risk factor for CSC [8]. Glucocorticoids bind to the GR and to the MR. Both GR and MR are expressed in the retina and in the choroid vessels, demonstrating that the retina and the choroid are novel targets for steroid therapy [11]. Activation of the MR in the choroid by steroids causes choroid vessel dilation and leakage, leading to choroidal hyperpermeability that is known to be a major pathogenic factor in CSC [11]. This steroid-induced choroidal hyperpermeability can be blocked by MR antagonists [11]. Therefore, MR blockers are expected to have a therapeutic effect on CSC, and many studies have already shown good therapeutic efficacy of these drugs in treating CSC [12–18].

CSC may be caused by a variety of factors, but steroid-induced CSC is directly caused by steroids; we expected that MR antagonists may be more effective against this condition. In this current study, as expected, the MR antagonist showed good therapeutic effect in patients with steroid-induced CSC. Therefore, we considered that MR antagonists could be an important therapeutic option for patients with steroid-induced CSC who cannot discontinue steroid use. However, as seen in the present study result, when the MR antagonist was discontinued, SRF recurred. Continuous use of an MR antagonist should be considered during systemic steroid therapy in patients with steroid-induced CSC to prevent recurrence. Further studies are required on the efficacy and safety of long-term use of MR antagonists in steroid-induced CSC patients.

One of the major hurdles in using spironolactone for CSC treatment is systemic side effects. Spironolactone is a potassium-preserving diuretic and may cause medical side effects such as electrolyte abnormality [20]. To assess the presence of systemic side effects, we performed serum electrolyte examinations before treatment and every month during treatment. We found no significant changes in serum potassium or creatinine levels after spironolactone treatment (Table 2). There were no cases in which the drug had to be discontinued because of an electrolyte imbalance or spironolactone-related side effect. Therefore, this study showed that spironolactone used for CSC treatment produced fewer systemic side effects.

Our present study had several limitations due to its retrospective design. The sample size was also relatively small, which may have limited the statistical strength of the analysis. And, symptom duration of included patients was relatively short (4.00 ± 3.04 months). Therefore, it is possible that some of the improvement that was documented would have happened spontaneously. However, since the patients included in the study were not idiopathic CSC patients but CSC patients who were taking steroids, we thought that the probability of self-improvement was relatively low.

Despite these limitations, the current study provides new insights into the therapeutic effects of a MR antagonist in treating steroid-induced CSC. This study demonstrated that MR antagonist treatment, which blocks steroids from binding to the MR, was effective in treating steroid-induced CSC. In conclusion, MR antagonist treatment may be a therapeutic option for steroid-induced CSC patients. This treatment modality may be especially beneficial for steroid-induced CSC patients who cannot discontinue steroid medication because of systemic conditions.

## Figures and Tables

**Table 1:** Demographics of included patients.

Number of patients (eyes)	15 (17)
Age (years)	49.24 ± 7.70
Sex (male/female)	10/5
Symptom duration (months)	4.00 ± 3.04
Follow-up duration (months)	17.09 ± 6.40
Steroid dose (mg)	15.59 ± 5.58
Steroid use duration (months)	8.94 ± 9.03
Spironolactone dose (mg)	50
Spironolactone use duration (months)	2.59 ± 1.19
Underlying disease	
Myasthenia gravis (%)	8 (47.1)
Glomerular nephritis (%)	5 (29.4)
After organ transplantation (%)	4 (23.5)

**Table 2:** Serum level changes of potassium and creatinine after spironolactone treatment.

	Before spironolactone	After spironolactone	*P* value^*∗*^
Serum potassium	4.85 ± 0.47	4.91 ± 0.45	0.153
Serum creatinine	1.21 ± 0.35	1.30 ± 0.41	0.468

^*∗*^Paired *t*-test.

## Data Availability

The data used to support the findings of this study are included within the article.

## Additional Points

*Summary Statement.* Treatment of steroid-induced central serous chorioretinopathy (CSC) patients who cannot avoid steroid use could be challenging. This study showed that mineralocorticoid receptor (MR) antagonist treatment, which blocks steroids from binding to the MR, could be a good therapeutic option in steroid-induced CSC patients who cannot discontinue steroid therapy.
